# Comparison of antidiabetic drugs added to sulfonylurea monotherapy in patients with type 2 diabetes mellitus: A network meta-analysis

**DOI:** 10.1371/journal.pone.0202563

**Published:** 2018-08-27

**Authors:** Dan Qian, Tiantian Zhang, Xiangping Tan, Peiying Zheng, Zhuoru Liang, Jingmei Xie, Jie Jiang, Bing Situ

**Affiliations:** 1 Department of Pharmacy, The Third Affiliated Hospital of Guangzhou Medical University, Guangzhou, China; 2 College of Pharmacy, Jinan University, Guangzhou, China; University of Ioannina Medical School, GREECE

## Abstract

**Aims:**

This study aimed to investigate the efficacy and safety of dual therapy comprising sulfonylurea (SU) plus antidiabetic drugs for the treatment of type 2 diabetes mellitus (T2DM).

**Methods:**

We searched the PubMed, Cochrane library, and Embase databases for randomized clinical trials (≥24 weeks) published up to December 28, 2017. Subsequently, we conducted pairwise and network meta-analyses to calculate the odds ratios (ORs) and mean differences (MDs) with 95% confidence intervals (CIs) of the outcomes.

**Results:**

The final analyses included 24 trials with a total of 10,032 patients. Compared with placebo, all treatment regimens were associated with a significantly higher risk of hypoglycemia, except the combinations of SU plus sodium-glucose co-transporter-2 inhibitor (SGLT-2i) [OR, 1.35 (95% CI: 0.81 to 2.25)] or alpha-glucosidase inhibitor (AGI) [OR, 1.16 (95% CI: 0.55 to 2.44)]. Notably, the combination of SU plus glucagon-like peptide-1 receptor agonist (GLP-1RA) was associated with the most significant increase in the risk of hypoglycemia. Furthermore, all SU-based combination regimens reduced the glycated hemoglobin (HbA1c) and fasting plasma glucose levels (FPG). However, only combinations containing SGLT-2i [MD, -1.00 kg (95% CI: -1.73 to -0.27)] and GLP-1RA [MD, -0.56 kg (95% CI: -1.10 to -0.02)] led to weight loss.

**Conclusions:**

Our findings highlight the importance of considering the risk of hypoglycemia when selecting antidiabetic drugs to be administered concomitantly with SU. Although all classes of antidiabetic drugs improved glucose control when administered in combination with SU, SGLT-2i might be the best option with respect to factors such as hypoglycemia and body weight.

## Introduction

According to the most recent data from the International Diabetes Federation (IDF), the number of adults affected by diabetes worldwide reached 425 million in 2017, and approximately 4 million deaths were attributed to this disease. Current estimates suggest that 629 million people worldwide will be affected by diabetes in by 2045 [1]. Diabetes can be stratified into two types; of these, type 2 diabetes mellitus (T2DM) is a progressive disease characterized by initial insulin resistance and a subsequent decline in β cell function. Currently, metformin is the preferred therapeutic option for T2DM [2, 3]. However, metformin use is contraindicated for or not tolerated by some patients. In such cases, other hypoglycemic agents, including sodium-glucose co-transporter-2 inhibitors (SGLT-2is), dipeptidyl peptidase 4 inhibitors (DPP-4is), thiazolidinediones (TZDs), sulfonylureas (SUs), and alpha-glucosidase inhibitors (AGIs), may serve as first-line options [3]. SUs improve blood glucose by stimulating the β cells to secrete insulin in a non-glucose-dependent manner. Given the low costs and favorable efficacy and safety profiles, these drugs are used extensively following an initial diagnosis of T2DM, despite the potential to increase the incidence of hypoglycemic events and weight gain [4, 5].

As noted, however, β cell function declines over the course of T2DM, and most patients who initially used SUs will later require antidiabetic drug combination therapies to maintain glycemic control [6]. Several such SU-based combination regimens including: SGLT-2is, DPP-4is, TZDs, AGIs, glucagon-like peptide-1 receptor agonists (GLP-1RAs), and insulin are currently administered to patients. The process of selecting the appropriate second-line antidiabetic drug is complicated by various factors; in addition, the glycemic effects of the drugs, such as hypoglycemia and weight gain, must be considered because these can impact the patient's adherence to treatment and quality of life [7, 8].

With this work, we aimed to conduct a systematic review and network meta-analysis (NMA) of the most recently updated clinical trials to evaluate the efficacy and safety of antidiabetic drugs as add-on treatments for T2DM inadequately controlled with sulfonylurea monotherapy. Compared with the conventional pairwise meta-analysis method, the NMA enables us to calculate data from both direct and indirect comparisons of diverse regimens and to quantify and sort the efficacy and safety of each of these measures [9]. Using this approach, we hope to provide evidence to assist clinicians and patients with decision-making.

## Methods

The methods and results of this NMA have been reported in accordance with the PRISMA (Preferred Reporting Items for Systematic Reviews and Meta-Analyses) [10] recommendations and checklist (S1 Table).

### Search strategy

We searched the PubMed (from January 1, 1946 to December 28, 2017), Embase (from January 1, 1974 to December 28, 2017), and Cochrane library databases (issue 11 of 12, 2017) for relevant randomized clinical trials (RCTs). The following key terms were used: “SGLT-2 inhibitor”, “DPP-4 inhibitor”, “GLP-1”, “thiazolidinedione”, “alpha-glucosidase inhibitor”, “metformin” “insulin”, “diabetes mellitus”, and “randomized clinical trial”. More detailed search terms are listed in S2 Table. In addition, we checked the reference lists of all identified articles for other eligible studies. Additionally, we conducted a search of ClinicalTrials.gov. No time or language exclusion criteria were applied.

### Study selection and data extraction

Included studies were required to meet the following criteria: (1) RCT design; (2) study duration ≥24 weeks; (3) inclusion of adult (age: ≥18 years) patients with T2DM and inadequate glycemic control with sulfonylurea monotherapy; (4) drugs in the SGLT-2i, DPP-4i, GLP-1RA, TZD, AGI, metformin, and insulin classes; and (5) assessment of at least one of the following continuous outcomes—glycated hemoglobin (HbA1c), fasting plasma glucose (FPG), body weight—and dichotomous outcomes—hypoglycemia, serious adverse events (SAEs). SAEs were defined as fatal or life-threatening events, events requiring or extending inpatient hospitalization, those resulting in ongoing or significant incapacity or interfering substantially with normal life functions, and/or those that caused a congenital anomaly or birth defect. The primary outcome was hypoglycemia. Studies that met the following criteria were excluded: (1) patients with serious cardiovascular disease or severe renal impairment; (2) pregnant patients; (3) sample size <100; (4) non-randomized trials; and (5) publication as a conference report, letter, or abstract.

Two authors (D.Q. and T.Z.) independently assessed the titles and abstracts identified in the initial search and reviewed the full texts of all identified studies that met the inclusion criteria. The following details were recorded using a pre-defined spreadsheet: first author (publication year), study duration, interventions (types and doses), sample size, and baseline participant information (HbA1c, body mass index, body weight, age, sex). For studies with different follow-up durations, the longest reported duration was recorded. Any conflicts were resolved by the third author (P.Z.).

### Risk of bias

The Cochrane risk-of-bias tool [11] was used to assess the quality of the included RCTs. This tool assesses 7 domains, namely random allocation, allocation concealment, blinding of participants and personnel, blinding of outcome assessment, incomplete outcome data, selective reporting, and other biases. For each domain, the risk of bias was determined to be low, unclear, or high.

### Statistical analysis

In this study, the results of comparisons of dichotomous (hypoglycemia, SAEs) and continuous variables (HbA1c, FPG, and body weight) are reported as odds ratios (ORs) and mean differences (MDs), respectively, together with the corresponding 95% confidence intervals (CI).

A DerSimonian-Laird random effects model was used for the conventional pairwise meta-analysis [12]. Heterogeneity was evaluated using the I^2^ statistic, with an I^2^ of 25%, 50%, or 75% indicating low, moderate, or high heterogeneity, respectively [13]. For the NMA, a frequentist random-effects model was used with the assumption of a common between-study covariance structure across the treatment arms; this is often referred to as a homogenous variance assumption [14, 15]. In such a model, we included only 2 arms from trials including 3 overlapping arms (e.g., only A versus B from a trial with A versus B versus A + B). In addition, if the same drug was evaluated at different doses in the trials, we combined different doses into a single dose. For this analysis, we used the mvmeta and network commands in Stata software (Stata Corp, College Station, TX, USA) and the programmed Stata routines [16, 17]. To rank the probabilities of each intervention in terms of efficacy and safety outcomes, we used surface under the cumulative ranking (SUCRA) curves and mean ranks. Higher SUCRA values indicate better efficacy or safety [18]. A 0.5 zero-cell correction was applied when studies reported zero events [19]. The heterogeneity variance (tau [τ]) was estimated using a restricted maximum likelihood method and employed to estimate heterogeneity [20]. For the purposes of this analysis, we assumed that common methods of comparing glucose-lowering strategies were used similarly when reported by different trials and that participants included in those trials could be randomly allocated to any of the compared treatments.

To check for possible inconsistency, a loop-specific approach was used to evaluate differences between the direct and indirect estimates in each closed triangular or quadrangular loop [21]. Additionally, a “design-by-treatment” interaction model was applied to evaluate global heterogeneity within networks [22]. The small-study effects of active treatments versus placebo were assessed using comparison-adjusted funnel plots [23].

All P-values were 2-tailed, and P values of <0.05 were considered statistically significant. All analyses were performed using Stata 14.1 software.

## Results

### Characteristics of the included studies

**Fig 1:**
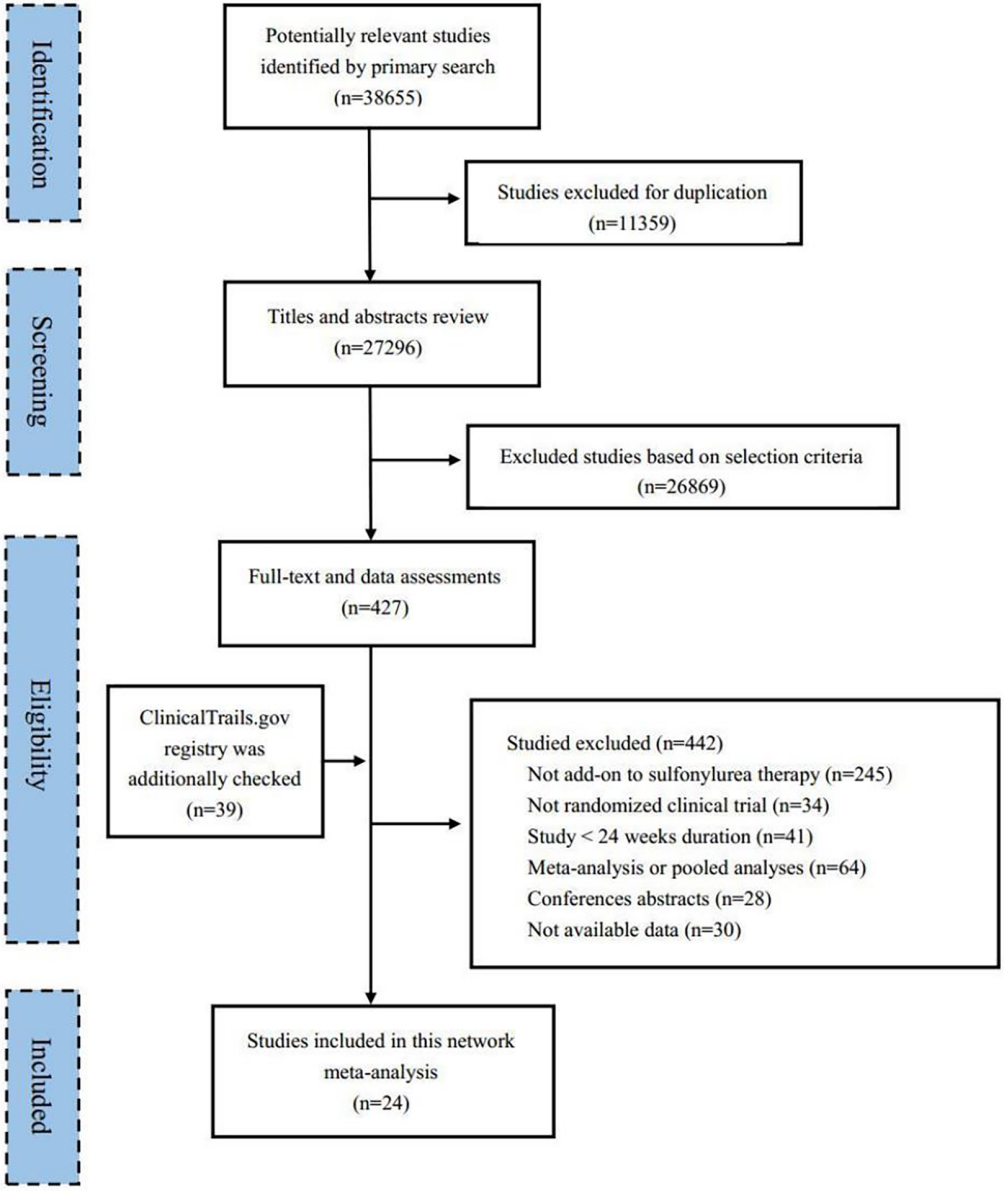
Flow chart of the search for eligible studies.

The PRISMA flow chart of this study is shown in Fig 1. A total of 38,655 articles were obtained from the searched databases, from which 427 potentially relevant articles were identified after de-duplication and abstract screening. Trials not involving add-on sulfonylurea therapy (245), those with a non-RCT design (34) or a study duration of <24 weeks (41), meta-analyses and pooled analyses (64), conference abstracts (28), and studies with no available data (30) were excluded. Finally, 24 eligible studies satisfied the inclusion criteria and were included in this NMA.

The included studies were published from 2000 to 2017 and had enrolled 10,032 (range, 105–1041) participants with T2DM. All 24 RCTs [24–47] reported data for follow-ups ranging from 24 to 52 weeks’ duration, while 2 [46, 47] additionally reported data between 52and 104 weeks. The mean [range] baseline HbA1c was 8.5% [7.6–9.9%], the mean ages ranged from 52 to 75 years, the mean [range] baseline BMI was 28.6 [23.8–32.2] kg/m^2^, and the mean [range] baseline weight was 78.1 [62.6–99] kg. Table 1 summarizes the detailed information from the included studies. The baseline characteristics of participants in these studies were deemed sufficiently similar in terms of age, sex, HbA1c, body weight, and body mass index (BMI) to permit network comparison (S1 Fig). There were also no specific clinical reasons (based on the inclusion and exclusion criteria of every trial in the network) to suggest that the type of participants under one comparison would be different from the type of participants in other comparisons. The S3–S6 Tables detail the numbers of participants included in the efficacy and safety outcome analyses by study and drug class.

**Table 1:** Characteristics of the included studies.

Author, year	Interventions	Sample size	Mean Age (years)	Male (%)	Mean HbA1c (%)	Mean body weight, kg	Mean BMI (kg/m^2^)	Study duration (weeks)
Ba 2017^[24]^	Sita: 100mg	249	57.5	47.0	8.6	68.4	25.4	24
	PLA	249	56.5	53.0	8.5	68.9	25.3	24
Gantz 2017^[25]^	Omar: 25mg	126	63	72.2	8.1	65	24.5	24
	PLA	63	63	71.4	8.1	67	24.8	24
Yang 2015^[26]^	Vild: 50mg	143	58.3	55.2	8.6	67.4	24.8	24
	PLA	136	58.7	58.1	8.7	68.8	25.0	24
Hermansen 2007^[27]^	Sita: 100mg	222	55.6	52.7	8.3	86.5	31.2	24
	PLA	219	56.5	53.4	8.3	85.9	30.7	24
Barnett 2013^[28]^	Lina: 5mg	95	75	72	7.8	86.3	29.6	24
	PLA	43	75*	62*	7.7*	86.4*	29.8*	24
Garber 2008^[29]^	Vild: 50mg	132	58.6	78	8.5	NA	32.2	24
	Vild: 100mg	132	58.2	79	8.6	NA	30.8	24
	PLA	144	57.9	84	8.5	NA	31.0	24
Pratley 2009^[30]^	Alog: 12.5mg	203	56.5	54.7	NA	NA	30.2	26
	Alog: 25mg	198	56.5	50.0	NA	NA	30.0	26
	PLA	99	57.1	51.5	NA	NA	30.0	26
Chacra 2009^[31]^	Saxa: 2.5mg	248	55.4	45.6	8.4	75.2	29.1	24
	Saxa: 5mg	253	54.9	43.5	8.5	76.2	29.2	24
	PLA	267	55.1	46.1	8.4	75.6	28.8	24
Yale 2017^[32]^	Cana: 100mg	74	65.8	50.0	8.3	80.7	NA	52
	Cana: 300mg	72	64.3	58.3	8.1	80.5	NA	52
	PLA	69	64.3	59.4	8.4	84.2	NA	52
Dungan 2016^[33]^	Dula: 1.5mg	239	57.7	43.5	8.4	84.5	30.9	24
	PLA	60	58.2	46.7	8.4	89.5	32.4	24
Forst 2015^[34]^	Vild: 50mg	82	65.9	56.1	7.6	85.5	29.7	24
	NPH insulin	79	67.6	60.8	7.7	89.9	31.4	24
Strojek 2014^[35]^	Dapa: 2.5mg	154	59.9	50.0	8.1	81.9	30.0	48
	Dapa: 5mg	142	60.2	50.0	8.1	81.0	29.8	48
	Dapa: 10mg	151	58.9	43.7	8.1	80.6	29.8	48
	PLA	145	60.3	49.0	8.2	80.9	29.7	48
Hsieh 2011^[36]^	Migi: 50mg	52	58.4	44.2	8.1	67.2	25.3	24
	PLA	53	59.0	34.0	8.1	69.2	26.1	24
Scheen 2009^[37]^	Piog: <45mg	508	63.2	67.9	7.8	NA	29.7	30
	PLA	493	62.9	70.6	7.7	NA	29.9	30
Marre 2009^[38]^	Lira: 0.6mg	233	55.7	54	8.4	82.6	30.0	26
	Lira: 1,2mg	228	57.7	45	8.5	80.0	29.8	26
	Lira: 1.8mg	234	55.6	53	8.5	83.0	30.0	26
	Rosi: 4mg	232	56.0	47	8.4	80.6	29.4	26
	PLA	114	54.7	47	8.4	81.9	30.3	26
Davidson 2007^[39]^	Rosi: 8mg	117	52	54.7	9.2	86.3	31.3	24
	PLA	116	53	51.7	9.4	88.3	31.9	24
Buse 2004^[40]^	Exen: 5μg	125	55	59.2	8.5	95	33	30
	Exen: 10μg	129	56	57.4	8.6	95	33	30
	PLA	123	55	62.6	8.7	99	34	30
Araki 2015^[41]^	Empa: 10mg	136	61.3	72.8	8.0	65.8	24.6	52
	Empa: 25mg	137	61.8	70.1	8.1	67.0	25.2	52
	Met: <2550mg	63	60.0	74.6	7.9	68.2	25.2	52
Kobayashi 2014^[42]^	Sita: 50mg	59	64.3	61.0	7.7	62.6	23.8	24
	Migi: 50mg	55	64.0	61.8	7.6	65.0	24.7	24
Wolffenbuttel 2000^[43]^	Rosi: 2mg	199	61.0	62.8	9.2	NA	28.0	26
	Rosi: 4mg	183	60.6	55.2	9.2	NA	28.3	26
	PLA	192	61.9	57.3	9.2	NA	28.1	26
Kaku 2010^[44]^	Lira: 0.6mg	88	59.1	60	8.6	66.1	25.3	24
	Lira: 0.9mg	88	61.3	67	8.2	64.5	24.4	24
	PLA	88	58.6	65	8.4	66.7	24.9	24
Zhu 2003^[45]^	Rosi: 4mg	215	59.0	41	9.8	NA	24.8	24
	Rosi: 8mg	210	58.9	48	9.9	NA	24.9	24
	PLA	105	58.8	46	9.8	NA	25.1	24
Seufert 2008^[46]^	Piog: <45mg	319	60	53.6	8.8	NA	30.2	104
	Met: <2550mg	320	60	54.7	8.8	NA	30.0	104
Bachmann 2003^[47]^	Acar: 100mg	164	63.8	52.4	9.4	80.7	29.0	78
	PLA	166	63.3	56.6	9.4	81.6	29.0	78

* Data refer to the overall study population.

HbA1c, glycated hemoglobin; BMI, body mass index; Alog, alogliptin; Saxa: saxagliptin; Vild, vildagliptin; Lina, linagliptin; Sita, sitagliptin; Omar, omarigliptin; Lira, liraglutide; Exen, exenatide; Dula, Dulaglutide; Acar, acarbose; Migi, miglitol; Piog, pioglitazone; Rosi, rosiglitazone; Met, metformin; PLA, placebo; NA, not applicable.

The Cochrane system bias evaluation is shown in S7 Table and S2 Fig. All studies were randomized and double blinded, and the risks of bias for random sequence generation, concealment of treatment allocation, and blinding of participants and personnel were low or unclear. Three studies had a high risk of reporting bias, of which 1 presented a high risk of detection bias. One study had incomplete outcome data.

### Network consistency

**Fig 2:**
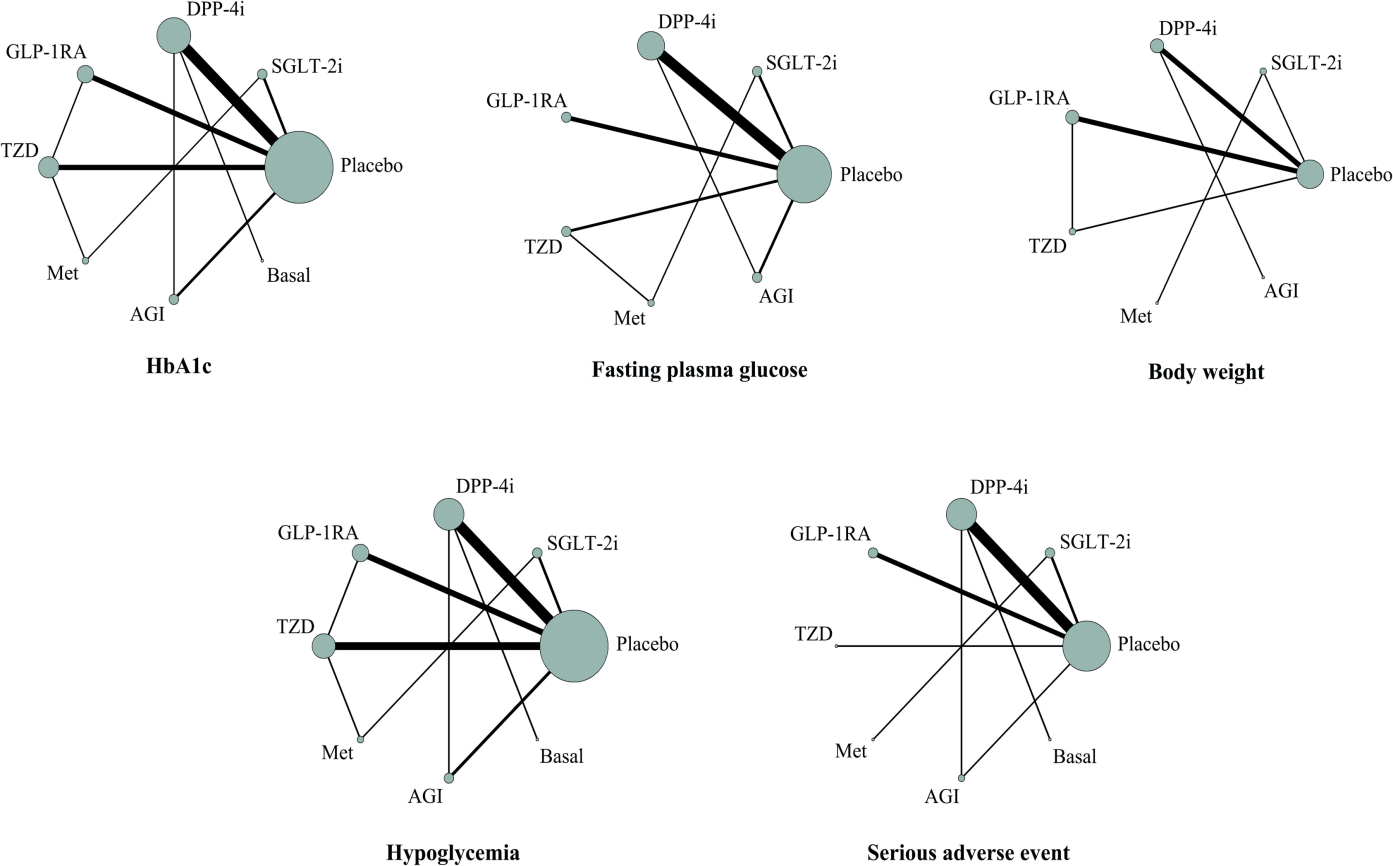
Network maps for efficacy and safety outcomes. Note: Connecting lines represent direct comparisons between the pairs of treatments, and the line widths represent the numbers of trials. The node sizes represent the overall sample sizes of the interventions. HbA1c, glycated hemoglobin; FPG, fasting plasma glucose; SGLT-2i, sodium-glucose co-transporter-2 inhibitor; DPP-4i, dipeptidyl peptidase-4 inhibitor; GLP-1RA, glucagon-like peptide-1 receptor agonist; TZD, thiazolidinedione; Met, metformin; AGI, α-glucosidase inhibitor; Basal, basal (long-acting) insulin.

The networks of eligible comparisons of efficacy and safety outcomes are graphically displayed in Fig 2. There were no loop inconsistencies between the evidence derived from direct and indirect comparisons for the 95% CIs of the IF values including zero values (S8 Table). In addition, the design-by-treatment model did not detect global inconsistency within any network (p for all >0.05, S9 Table). The contribution of each study to NMA is shown in S10 Table.

### Primary outcome: Hypoglycemia

**Fig 3:**
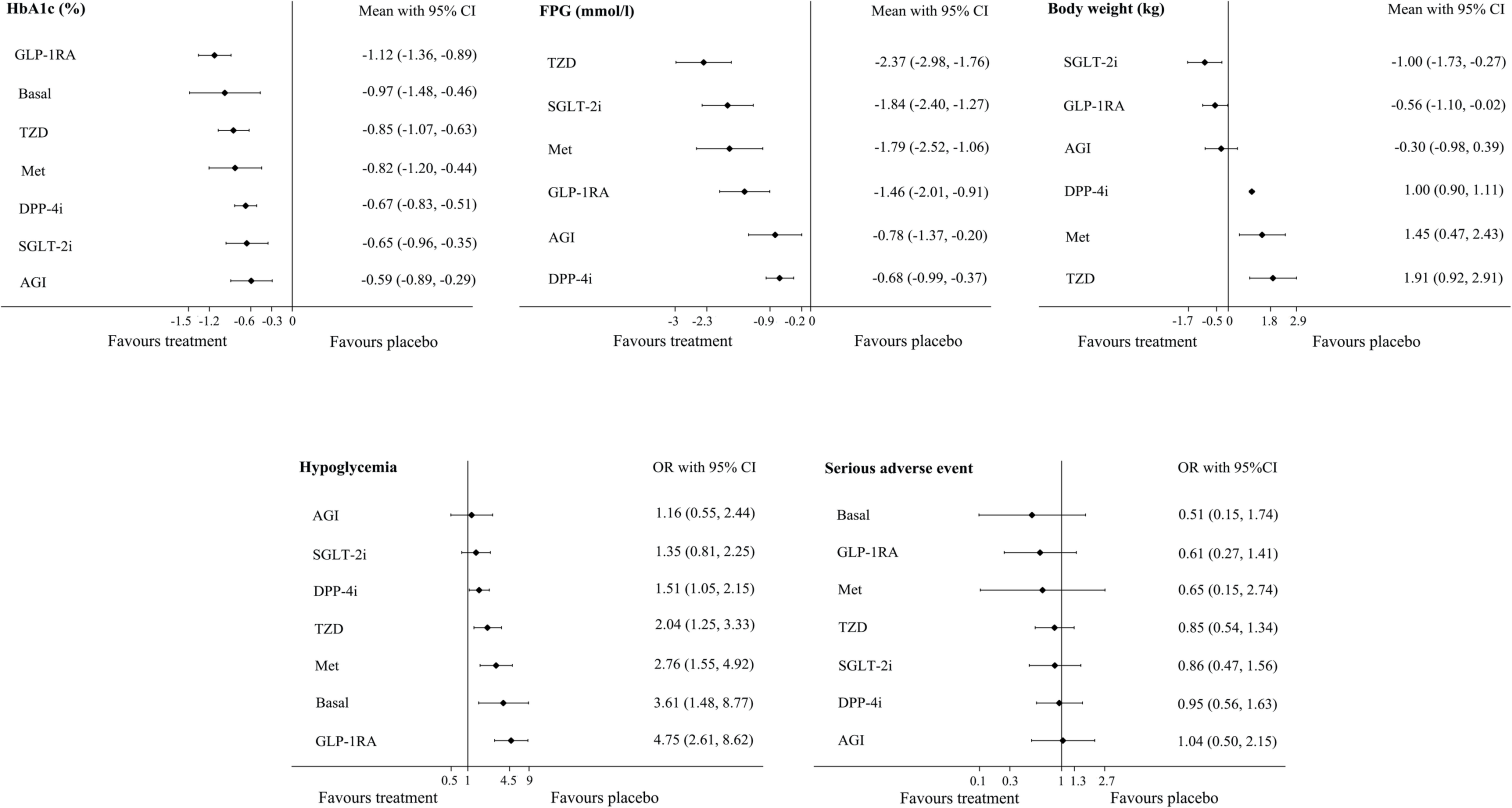
Differences versus placebo in efficacy and safety outcomes. Note: HbA1c, glycated hemoglobin; FPG, fasting plasma glucose; SGLT-2i, sodium-glucose co-transporter-2 inhibitor; DPP-4i, dipeptidyl peptidase-4 inhibitor; GLP-1RA, glucagon-like peptide-1 receptor agonist; TZD, thiazolidinedione; Met, metformin; AGI, α-glucosidase inhibitor; Basal, basal (long-acting) insulin.

The analysis of hypoglycemia included data from 23 RCTs including 9486 participants; of these, 939 participants had reported events (S3 Table). A direct pairwise meta-analyses showed an increased risk of hypoglycemia with GLP-1RA [OR, 7.56 (95% CI: 3.44 to 16.59)], TZD [OR, 2.33 (95% CI: 1.26 to 4.32)], and DPP-4i [OR, 1.54 (95% CI: 1.10 to 2.15)] (S11 Table). The NMA results showed that DPP-4i, TZD, Met, basal insulin, and GLP-1RA were more strongly associated with hypoglycemia, compared with placebo. No significant differences relative to placebo were observed for SGLT-2i [OR, 1.35 (95% CI: 0.81 to 2.25)] and AGI [OR, 1.16 (95% CI: 0.55 to 2.44)]. Compared with GLP-1RA, all agents were associated with a lower risk of hypoglycemia, except Met [OR, 0.58 (95% CI: 0.29 to 1.18)] and basal insulin [OR, 0.76 (95% CI: 0.27 to 2.17)], which appeared to have no significant effects on the risk of hypoglycemia (Table 2 and Fig 3). Excluding placebo, AGI and SGLT-2i most strongly reduced the risk of hypoglycemia, with SUCRA values of 79% and 71.7%, respectively. The lowest SUCRA value was calculated for GLP-1RA (5.2%) (Table 3).

**Table 2:** Effect of glucose-lowering agents in patients with type 2 diabetes.

**Hypoglycemia (OR, 95% CI)**							
**PLA**	1.35 (0.81, 2.25)	**1.51** **(1.05, 2.15)**	**4.75** **(2.61, 8.62)**	**2.04** **(1.25, 3.33)**	**2.76** **(1.55, 4.92)**	1.16 (0.55, 2.44)	**3.61** **(1.48, 8.77)**
0.74 (0.44, 1.24)	**SGLT-2i**	1.12 (0.60, 2.08)	**3.52** **(1.65, 7.54)**	1.52 (0.78, 2.93)	**2.05** **(1.05, 4.02)**	0.86 (0.35, 2.10)	2.68 (0.96, 7.45)
**0.66** **(0.46, 0.95)**	0.89 (0.48, 1.66)	**DPP-4i**	**3.15** **(1.63, 6.11)**	1.36 (0.78, 2.36)	1.83 (0.95, 3.54)	0.77 (0.35, 1.68)	2.40 (1.06, 5.40)
**0.21** **(0.12, 0.38)**	**0.28** **(0.13, 0.61)**	**0.32** **(0.16, 0.62)**	**GLP-1RA**	**0.43** **(0.25, 0.74)**	0.58 (0.29, 1.18)	**0.24** **(0.10, 0.59)**	0.76 (0.27, 2.17)
**0.49** **(0.30, 0.80)**	0.66 (0.34, 1.28)	0.74 (0.42, 1.29)	**2.33** **(1.35, 4.01)**	**TZD**	1.35 (0.81, 2.25)	0.57 (0.26, 1.24)	1.77 (0.66, 4.73)
**0.36** **(0.20, 0.65)**	**0.49** **(0.25, 0.96)**	0.55 (0.28, 1.05)	1.72 (0.84, 3.50)	0.74 (0.45, 1.23)	**Met**	0.42 (0.17, 1.03)	1.31 (0.46, 3.72)
0.86 (0.41, 1.83)	1.16 (0.48, 2.85)	1.30 (0.60, 2.84)	**4.10** **(1.70, 9.88)**	1.77 (0.81, 3.86)	2.39 (0.98, 5.84)	**AGI**	**3.12** **(1.01, 9.63)**
**0.28** **(0.11, 0.67)**	0.37 (0.13, 1.04)	0.42 (0.19, 0.94)	1.32 (0.46, 3.75)	0.57 (0.21, 1.52)	0.77 (0.27, 2.18)	**0.32** **(0.10, 0.99)**	**Basal**
**HbA1c, % (MD, 95% CI)**							
**PLA**	**-0.65** **(-0.96, -0.35)**	**-0.67** **(-0.83, -0.51)**	**-1.12** **(-1.36, -0.89)**	**-0.85** **(-1.07, -0.63)**	**-0.82** **(-1.20, -0.44)**	**-0.59** **(-0.89, -0.29)**	**-0.97** **(-1.48, -0.46)**
**0.65** **(0.35, 0.96)**	**SGLT-2i**	-0.02 (-0.36, 0.32)	**-0.47** **(-0.85, -0.09)**	-0.19 (-0.54, 0.15)	-0.17 (-0.53, 0.19)	0.06 (-0.36, 0.49)	-0.32 (-0.91, 0.27)
**0.67** **(0.51, 0.83)**	0.02 (-0.32, 0.36)	**DPP-4i**	**-0.45** **(-0.73, -0.16)**	-0.17 (-0.45, 0.10)	-0.15 (-0.56, 0.26)	0.08 (-0.23, 0.39)	-0.30 (-0.78, 0.18)
**1.12** **(0.89, 1.36)**	**0.47** **(0.09, 0.85)**	**0.45** **(0.16, 0.73)**	**GLP-1RA**	0.27 (-0.02, 0.56)	0.30 (-0.13, 0.73)	**0.53** **(0.15, 0.91)**	0.15 (-0.41, 0.71)
**0.85** **(0.63, 1.07)**	0.19 (-0.15, 0.54)	0.17 (-0.10, 0.45)	-0.27 (-0.56, 0.02)	**TZD**	0.02 (-0.34, 0.39)	0.26 (-0.12, 0.63)	-0.13 (-0.68, 0.43)
**0.82** **(0.44, 1.20)**	0.17 (-0.19, 0.53)	0.15 (-0.26, 0.56)	-0.30 (-0.73, 0.13)	-0.02 (-0.39, 0.34)	**Met**	0.23 (-0.25, 0.72)	-0.15 (-0.79, 0.49)
**0.59** **(0.29, 0.89)**	-0.06 (-0.49, 0.36)	-0.08 (-0.39, 0.23)	**-0.53** **(-0.91, -0.15)**	-0.26 (-0.63, 0.12)	-0.23 (-0.72, 0.25)	**AGI**	-0.39 (-0.97, 0.18)
**0.97** **(0.46, 1.48)**	0.32 (-0.27, 0.91)	0.30 (-0.18, 0.78)	-0.15 (-0.71, 0.41)	0.13 (-0.43, 0.68)	0.15 (-0.49, 0.79)	0.38 (-0.19, 0.96)	**Basal**
**Fasting plasma glucose, mmol/l (MD, 95% CI)**							
**PLA**	**-1.84** **(-2.40, -1.27)**	**-0.68** **(-0.99, -0.37)**	**-1.46** **(-2.01, -0.91)**	**-2.37** **(-2.98, -1.76)**	**-1.79** **(-2.52, -1.06)**	**-0.78** **(-1.37, -0.20)**	-
**1.84** **(1.27, 2.40)**	**SGLT-2i**	**1.15** **(0.51, 1.80)**	0.38 (-0.42, 1.17)	-0.53 (-1.25, 0.19)	0.04 (-0.61, 0.69)	**1.05** **(0.23, 1.87)**	-
**0.68** **(0.37, 0.99)**	**-1.15** **(-1.80, -0.51)**	**DPP-4i**	**-0.78** **(-1.41, -0.15)**	**-1.69** **(-2.37, -1.01)**	**-1.11** **(-1.90, -0.32)**	-0.10 (-0.72, 0.51)	-
**1.46** **(0.91, 2.01)**	-0.38 (-1.17, 0.42)	**0.78** **(0.15, 1.41)**	**GLP-1RA**	**-0.91** **(-1.73, -0.08)**	-0.33 (-1.25, 0.59)	0.67 (-0.13, 1.48)	-
**2.37** **(1.76, 2.98)**	0.53 (-0.19, 1.25)	**1.69** **(1.01, 2.37)**	0.91 (0.08, 1.73)	**TZD**	0.58 (-0.10, 1.26)	**1.58** **(0.74, 2.43)**	-
**1.79** **(1.06, 2.52)**	-0.04 (-0.69, 0.61)	**1.11** **(0.32, 1.90)**	0.33 (-0.59, 1.25)	-0.58 (-1.26, 0.10)	**Met**	**1.01** **(0.07, 1.95)**	-
**0.78** **(0.20, 1.37)**	**-1.05** **(-1.87, -0.23)**	0.10 (-0.51, 0.72)	-0.67 (-1.48, 0.13)	**-1.58** **(-2.43, -0.74)**	**-1.01** **(-1.95, -0.07)**	**AGI**	-
**Body weight, kg (MD, 95% CI)**							
**PLA**	**-1.00** **(-1.73, -0.27)**	**1.00** **(0.90, 1.11)**	**-0.56** **(-1.10, -0.02)**	**1.91** **(0.92, 2.91)**	**1.45** **(0.47, 2.43)**	-0.30 (-0.98, 0.39)	-
**1.00** **(0.27, 1.73)**	**SGLT-2i**	**2.00** **(1.27, 2.74)**	0.44 (-0.47, 1.35)	**2.91** **(1.68, 4.15)**	**2.45** **(1.80, 3.10)**	0.70 (-0.30, 1.71)	-
**-1.00** **(-1.11, -0.90)**	**-2.00** **(-2.74, -1.27)**	**DPP-4i**	**-1.56** **(-2.11, -1.02)**	0.91 (-0.09, 1.91)	0.45 (-0.54, 1.43)	**-1.30** **(-1.98, -0.62)**	-
**0.56** **(0.02, 1.10)**	-0.44 (-1.35, 0.47)	**1.56** **(1.02, 2.11)**	**GLP-1RA**	**2.47** **(1.61, 3.34)**	**2.01** **(0.89, 3.12)**	0.26 (-0.61, 1.14)	-
**-1.91** **(-2.91, -0.92)**	**-2.91** **(-4.15, -1.68)**	-0.91 (-1.91, 0.09)	**-2.47** **(-3.34, -1.61)**	**TZD**	-0.46 (-1.86, 0.93)	**-2.21** **(-3.42, -1.00)**	-
**-1.45** **(-2.43, -0.47)**	**-2.45** **(-3.10, -1.80)**	-0.45 (-1.43, 0.54)	**-2.01** **(-3.12, -0.89)**	0.46 (-0.93, 1.86)	**Met**	**-1.75** **(-2.94, -0.55)**	-
0.30 (-0.39, 0.98)	-0.70 (-1.71, 0.30)	**1.30** **(0.62, 1.98)**	-0.26 (-1.14, 0.61)	**2.21** **(1.00, 3.42)**	**1.75** **(0.55, 2.94)**	**AGI**	-
**Serious adverse event (OR, 95% CI)**							
**PLA**	0.86 (0.47, 1.56)	0.95 (0.56, 1.63)	0.61 (0.27, 1.41)	0.85 (0.54, 1.34)	0.65 (0.15, 2.74)	1.04 (0.50, 2.15)	0.51 (0.15, 1.74)
1.16 (0.64, 2.11)	**SGLT-2i**	1.11 (0.49, 2.52)	0.71 (0.25, 2.04)	0.99 (0.47, 2.10)	0.75 (0.20, 2.80)	1.21 (0.47, 3.10)	0.59 (0.15, 2.35)
1.05 (0.61, 1.79)	0.90 (0.40, 2.04)	**DPP-4i**	0.64 (0.25, 1.67)	0.89 (0.44, 1.80)	0.68 (0.14, 3.19)	1.09 (0.45, 2.63)	0.53 (0.17, 1.61)
1.63 (0.71, 3.76)	1.40 (0.49, 4.02)	1.56 (0.60, 4.07)	**GLP-1RA**	1.39 (0.54, 3.59)	1.06 (0.20, 5.69)	1.69 (0.56, 5.12)	0.83 (0.19, 3.59)
1.17 (0.75, 1.84)	1.01 (0.48, 2.13)	1.12 (0.56, 2.25)	0.72 (0.28, 1.85)	**TZD**	0.76 (0.17, 3.44)	1.21 (0.51, 2.87)	0.59 (0.16, 2.21)
1.55 (0.37, 6.54)	1.33 (0.36, 4.94)	1.47 (0.31, 6.94)	0.95 (0.18, 5.09)	1.32 (0.29, 5.98)	**Met**	1.60 (0.32, 8.08)	0.78 (0.12, 5.27)
0.97 (0.46, 2.01)	0.83 (0.32, 2.13)	0.92 (0.38, 2.23)	0.59 (0.20, 1.78)	0.82 (0.35, 1.94)	0.62 (0.12, 3.15)	**AGI**	0.49 (0.12, 2.02)
1.98 (0.58, 6.78)	1.70 (0.43, 6.76)	1.88 (0.62, 5.73)	1.21 (0.28, 5.25)	1.68 (0.45, 6.27)	1.28 (0.19, 8.60)	2.05 (0.49, 8.47)	**Basal**

For the lower triangle, comparisons should be read from left to right. Odds ratios (ORs) <1 favor the column-defining treatment and mean differences (MDs) <0 favor the column-defining treatment. For the upper triangle, comparisons should be read from right to left. Odds ratios (ORs) <1 favor the row-defining treatment and mean differences (MDs) <0 favor the row-defining treatment. Bold fonts indicate statistically significant differences. HbA1c, glycated hemoglobin; SGLT-2i, sodium-glucose co-transporter-2 inhibitor; DPP-4i, dipeptidyl peptidase-4 inhibitor; GLP-1RA, glucagon-like peptide-1 receptor agonist; TZD, thiazolidinedione; Met, metformin; AGI, α-glucosidase inhibitor; Basal, basal (long-acting) insulin, PLA, placebo.

**Table 3:** The results of the surface under the cumulative ranking curve (SUCRA) ranking probabilities.

Treatment	HbA1c		FPG		Body weight		Hypoglycemia		SAE	
	SUCRA	Rank	SUCRA	Rank	SUCRA	Rank	SUCRA	Rank	SUCRA	Rank
Placebo	0	8	0.1	7	53.7	4	93.4	1	29.1	8
SGLT-2i	36.7	6	74.3	2	95.7	1	71.7	3	47.3	5
DPP-4i	39.4	5	22.6	6	29.4	5	63.7	4	36.3	7
GLP-1RA	94	1	56.1	4	81.8	2	5.2	8	70.5	2
TZD	66.1	3	97.8	1	5	7	43.7	5	47.8	4
Met	60.7	4	70.5	3	15.7	6	25.7	6	61	3
AGI	28.2	7	28.5	5	68.7	3	79	2	31.2	6
Basal	75	2	NA	NA	NA	NA	17.6	7	76.8	1
Heterogeneity(tau)^a^	0.21		0.35		≈0		0.11		0.19	

Larger SUCRAs and lower median ranks indicate better treatments (i.e. SUCRA values of 100% and 0% correspond to the best and worst treatments, respectively).

^a^ Degree of between-study heterogeneity.

HbA1c, glycated hemoglobin; FPG, fasting plasma glucose; SAE, serious adverse event; SGLT-2i, sodium-glucose co-transporter-2 inhibitor; DPP-4i, dipeptidyl peptidase-4 inhibitor; GLP-1RA, glucagon-like peptide-1 receptor agonist; TZD, thiazolidinedione; Met, metformin; AGI, α-glucosidase inhibitor; Basal, basal (long-acting) insulin; NA, not applicable.

### Efficacy outcomes: HbA1c, FPG, and body weight

HbA1c data were available from 8930 patients in 23 RCTs (S4 Table). In direct pairwise meta-analyses, the largest HbA1c reduction was observed with GLP-1RA versus placebo [-1.05% (95% CI: -1.35 to -0.76)]. In comparisons of other antidiabetic drugs, the significant differences ranged from a reduction of -0.30% (95% CI: -0.55 to -0.05) for basal insulin versus DPP-4i to -0.49% (95% CI: -0.64 to -0.34) for GLP-1RA versus TZD (S12 Table). The NMA indicated that when compared to placebo, all drug classes were associated with greater reductions in HbA1c levels when combined with SU. Comparisons among the second-line agents in combination with SU revealed greater HbA1c reductions with GLP-1RA versus SGLT-2i [-0.47% (95% CI: -0.85 to -0.09)], DPP-4i [-0.45% (95% CI: -0.73 to -0.16)], and AGI [-0.53% (95% CI: -0.91 to -0.15)]. No significant differences were observed for the other comparisons between second-line agents combined with SU (Table 2 and Fig 3). Notably, GLP-1RA and basal insulin yielded the greatest reductions in HbA1c, with SUCRA values of 94% and 75%, respectively (Table 3).

Nineteen RCTs with a total of 6611 participants reported changes in FPG (S4 Table). Direct pairwise meta-analyses (versus placebo) showed significant reductions in FPG with all second-line agents combined with SU, ranging from -2.77 mmol/l (95% CI: -4.14 to -1.40) for TZD to -0.62 mmol/l (95% CI: -0.78 to -0.47) for DPP-4i. When compared with other antidiabetic drugs, the only significant difference was observed for SGLT-2i versus Met [-0.44 mmol/l (95% CI: -0.73 to -0.15)] (S12 Table). The NMA revealed that compared with placebo, all drug classes were associated with greater reductions in HbA1c levels when combined with SU (Table 2 and Fig 3). Among the second-line agents added to SU, DPP-4i was associated with the most significant increase in FPG versus all other agents, whereas AGI [0.10 mmol/l (95% CI: -0.51 to 0.72)] had no significant effect on the FPG levels (Table 2). TZD and SGLT-2i most significantly reduced the FPG level, with SUCRA values of 97.8% and 74.3%, respectively. Besides the placebo, DPP-4i received the lowest SUCRA value (22.6%) (Table 3).

Body weight values were available for 4516 participants in 11 RCTs (S4 Table). In direct pairwise meta-analyses of SU-containing combination regimens, the largest reduction in this parameter was observed for SGLT-2i versus placebo [-1.00 kg (95% CI: -1.73 to -0.27)], whereas the greatest increase was observed for DPP-4i versus placebo [1.00 kg (95% CI: 0.90 to 1.11)] (S12 Table). The NMA indicated reductions (versus placebo) of -1.00 kg (95% CI: -1.73 to -0.27) for SGLT-2i and -0.56 kg (95% CI: -1.10 to -0.02) for GLP-1RA. DPP-4i, TZD, and Met were found to increase body weight, whereas AGI had no significant effect on body weight (Table 2 and Fig 3). In comparisons of second-line agents, SGLT-2i, GLP-1RA, and AGI more significantly reduced body weight versus TZD, Met, and DPP-4i, with changes ranging from -2.91 kg (95% CI: -4.15 to -1.68) for SGLT–2i versus TZD to -1.30 kg (95% CI: -1.98 to -0.62) for AGI versus DPP-4i. No significant differences in body weight were observed between SGLT-2i, GLP-1RA, and AGI when added to SU (Table 2). SGLT-2i and GLP-1RA yielded the greatest reductions in body weight, with respective SUCRA values of 95.7% and 81.8% (Table 3).

### Other safety outcome: Serious adverse events

SAE data were available for 6335 participants in 17 RCTs (S3 Table). The NMA indicated no significant differences in the ORs of SAE for any agent when added to SU. Excluding placebo, basal insulin and AGI received the highest (76.8%) and lowest (36.2%) SUCRA values, respectively (Table 3).

### Publication bias

A comparison-adjusted funnel plot of all outcomes is displayed in S2 Fig. This analysis indicated no evidence of small-study effects with respect to active treatments versus placebo in the network.

## Discussion

Only 1 previous meta-analysis [48] assessed the risk of hypoglycemia of DPP-4i versus placebo when added to SU, and no head-to-head trials have estimated the relative effects of other antidiabetic drugs (especially SGLT-2i and GLP-1RA). To address this paucity of research, we performed the current NMA to combine high-quality data from the most updated trials and thus comprehensively compare the effects of SGLT-2i, DPP-4i, GLP-1RA, TZD, Met, AGI and basal insulin in patients with T2DM that was inadequately controlled by SU monotherapy. We hope that our findings will provide a comprehensive picture of the effects of these drugs when combined with SU.

Hypoglycemia is a serious clinical event that cannot be neglected during T2DM treatment. Related symptoms such as weakness, nervousness, trembling, and palpitations negatively affect the patient's quality of life and are closely associated with the risks of cardiovascular disease and hospital admission [49–51]. Additionally, even mild hypoglycemia can cause mental distress [52]. In our NMA, we found that when compared with placebo, all agents except SGLT-2i and AGI were associated with an increased risk of hypoglycemia when added to SU. Of these agents, GLP-1RA most significantly increased the risk of hypoglycemia relative to all other agents except Met and basal insulin and thus received the worst ranking. Our findings were consistent with the previous meta-analysis conducted by Salvo et al. [48], in which the combination of DPP-4i and SU was associated with a 50% increased risk of hypoglycemia versus placebo plus SU. However, we did not detect significant differences in the risks of hypoglycemia associated with SGLT-2i, DPP-4i, TZD, Met, or AGI plus SU. Generally, SGLT-2i reduces the risk of hypoglycemia, whereas SU drugs increase this risk. Although our results indicate that SGLT-2i and AGI did not cause statistically significant increases in the risk of hypoglycemia when added to SU, the SU dosage may need to be adjusted in such regimens to mitigate this risk [48].

Beta-cell dysfunction plays a vital role at all stages in the pathogenesis of T2DM, and these effects are compounded by insulin resistance [53, 54]. Our study demonstrated that all classes of antidiabetic drugs improved glucose control relative to the placebo (0.59–1.12% decrease in HbA1c and 0.68–2.37 mmol/l decrease in FPG) when combined with SU. Of these drugs, GLP-1RA was associated with the greatest reduction in HbA1c levels when added to SU monotherapy. Furthermore, no single agent other than GLP-1RA could significantly reduce HbA1c to a greater extent than any other when added to SU. However, only 2 RCTs included in our study reported durations >52 weeks [46, 47]. As HbA1c is a relatively stable variable used to reflect long-term glucose control, future research may be needed to estimate the long-term efficacies of these agents when combined with SU.

Weight gain has been suggested to correlate with an increased risk of diabetes, suggesting that weight control would be favorable for blood glucose control [55, 56]. Our NMA showed that compared to placebo, only SGLT-2i and GLP-1RA were associated with significant decreases in body weight when added to SU. Notably, SGLT-2i yielded significant improvements in this parameter when compared with all other agents except GLP-1RA and AGI and was therefore ranked the best. A previous NMA by Kay et al. [57] demonstrated that all DPP-4is were associated with mean body weight gains relative to placebo when added to metformin and SU. Although that study focused on triple combination therapy (DPP-4i + Met + SU), these findings were consistent with our study results.

To our knowledge, ours is the first NMA to compare the efficacy and safety profiles of all available agents in patients with T2DM inadequately controlled with SU monotherapy. The previous meta-analysis conducted by Salvo and his colleague [48] evaluated the effects of DPP-4i on the risk of hypoglycemia relative to placebo, rather than to other active agents. Therefore, we aimed to fill the gaps left by that study. We comprehensively estimated the relative effects of all available agents administered in combination with SU to provide additional information that would assist clinicians with decision-making. However, some potential limitations of our study should be considered. First, the majority of the RCTs had study durations of 24–52 weeks, while only 2 had follow-up durations >52 weeks. Obviously, therefore, our conclusions should be applied cautiously when evaluating the long-term effects of these antidiabetic drugs when combined with SU. Second, although we evaluated the effects of each class of antidiabetic drugs as a whole, some within-class differences were observed. Further studies might focus on the effects of different doses of these drugs when combined with SUs for T2DM. Third, the varying definitions of hypoglycemic events in the included studies may have contributed to clinical heterogeneity. Fourth, the quality of our analysis might have been compromised by inter-study differences in patient discontinuation rates. Fifth, we did not assess the baseline age, sex, HbA1c, body weight, BMI, diabetes duration, or duration of treatment, as effect modifiers when estimating efficacy and safety outcomes; the focus of further studies should be on evaluating the effects of these variables. Finally, only 1 RCT included data for basal insulin added to SU, which had a relatively wide confidence interval. This factor might have had some effect on our conclusions.

In conclusion, all classes of antidiabetic drugs improved glucose control when added to SU. However, SGLT-2i exhibited superior effects in terms of weight loss and did not increase the risk of hypoglycemia, suggesting that it might be the best option. Clinicians should particularly consider the risk of hypoglycemia when selecting antidiabetic drugs for administration together with SU.

## Supporting information

S1 Table PRISMA network meta-analysis checklist. (PDF)

S2 Table Search strategies. (PDF)

S3 Table Number of total participants and participants with events according to safety outcome and study. (PDF)

S4 Table Number of participants by efficacy outcome and study. (PDF)

S5 Table Numbers of arms, participants, and participants with events by safety outcome and drug. (PDF)

S6 Table Numbers of arms and participants by efficacy outcome and drug. (PDF)

S7 Table Assessment of the risk of bias in individual studies. (PDF)

S8 Table Assessment of the loop inconsistency within networks. (PDF)

S9 Table Assessment of global inconsistency within networks using the ‘design-by-treatment’ model. (PDF)

S10 Table Contributions of direct evidence in the entire network. (PDF)

S11 Table Pairwise random-effects meta-analyses of hypoglycemia and serious adverse events. (PDF)

S12 Table Pairwise random-effects meta-analyses of HbA1c, fasting plasma glucose, and body weight. (PDF)

S1 Fig The distribution of study-level characteristics according to drug class. (PDF)

S2 Fig Cochrane system bias evaluation chart of eligible studies. (PDF)

S3 Fig Comparison-adjusted funnel plot for efficacy and safety outcomes. (PDF)

S1 File Original data. (XLSX)
