# Supplementary material for: Comparison of antidiabetic drugs added to sulfonylurea monotherapy in patients with type 2 diabetes mellitus: A network meta-analysis
Source: PLoS One. 2018 Aug 27;13(8):e0202563. doi: 10.1371/journal.pone.0202563 (PMC6110472; doi:10.1371/journal.pone.0202563)
Supplement: S2 Table — (PDF) [file pone.0202563.s002.pdf]

S2 Table. Search Strategies

Data source	Search terms
**PubMed**	#1 Sodium glucose co-transporter 2<br>#2 SGLT2 OR SGLT-2 OR SGLT 2<br>#3 Tofogliflozin OR Empagliflozin OR Dapagliflozin OR Canagliflozin OR Sotagliflozin OR Luseogliflozin OR Ipragliflozin OR Remogliflozin OR Sergliflozin OR Ertugliflozin<br>#4 OR#1 - #3<br>#5 DPP-4 OR DDP4 OR DPP 4<br>#6 Vildagliptin OR Saxagliptin OR Sitagliptin OR Linagliptin OR Alogliptin OR Dutogliptin OR Gemigliptin OR Camegliptin OR Teneligliptin<br>#7 #5 OR #6<br>#8 Metformin<br>#9 Alpha glucosidase inhibitor OR Acarbose OR Miglitol OR Voglibose<br>#10 Glitazone OR Thiazolidinedione OR Pioglitazone OR Rivoglitazone OR Rosiglitazone<br>#11 GLP-1 OR Exenatide OR Liraglutide OR Lixisenatide OR Albiglutide OR Dulaglutide OR Semaglutide OR Taspoglutide<br>#12 Insulin OR Aspart OR Glulisine OR Lispro OR Glargine OR Degludec OR Detemir OR Isophane<br>#13 #4 OR #7 OR #8 OR #9 OR #10 OR #11 OR #12<br>#14 Diabetes mellitus or Diabetes<br>#15 random*<br>#16 "Randomized Controlled Trial"[Publication Type]<br>#17 RCT or RCTs<br>#18 OR/#15 - #17<br>#19 #13 AND #14 AND #18
**CENTRAL**	TITLE-ABSTRACT- KEYWORDS (Sodium Glucose co-transporter 2 OR SGLT2 OR SGLT-2 OR SGLT 2 OR Tofogliflozin OR Empagliflozin OR Dapagliflozin OR Canagliflozin OR Sotagliflozin OR luseogliflozin OR Ipragliflozin OR Remogliflozin OR Sergliflozin OR Ertugliflozin OR DPP-4 OR DDP4 OR DPP 4 OR Vildagliptin

	OR Saxagliptin OR Sitagliptin OR Linagliptin OR Alogliptin OR Dutogliptin OR Gemigliptin OR Camegliptin OR Teneligliptin OR Metformin OR GLP-1 OR Exenatide OR Liraglutide OR Lixisenatide OR Albiglutide OR Dulaglutide OR Semaglutide OR Taspoglutide OR Insulin OR Aspart OR Glulisine OR Lispro OR Glargine OR Degludec OR Detemir OR Isophane OR Alpha glucosidase inhibitor OR Acarbose OR Miglitol OR Voglibose OR Glitazone OR Thiazolidinedione OR Pioglitazone OR Rivoglitazone OR Rosiglitazone) AND (Diabetes mellitus or Diabetes)
**Embase**	TITLE-ABSTRACT-AUTHOR KEYWORDS (Sodium Glucose co-transporter 2 OR SGLT2 OR SGLT-2 OR SGLT 2 OR Tofogliflozin OR Empagliflozin OR Dapagliflozin OR Canagliflozin OR Sotagliflozin OR luseogliflozin OR Ipragliflozin OR Remogliflozin OR Sergliflozin OR Ertugliflozin OR DPP-4 OR DDP4 OR DPP 4 OR Vildagliptin OR Saxagliptin OR Sitagliptin OR Linagliptin OR Alogliptin OR Dutogliptin OR Gemigliptin OR Camegliptin OR Teneligliptin OR Metformin OR GLP-1 OR Exenatide OR Liraglutide OR Lixisenatide OR Albiglutide OR Dulaglutide OR Semaglutide OR Taspoglutide OR Insulin OR Aspart OR Glulisine OR Lispro OR Glargine OR Degludec OR Detemir OR Isophane OR Alpha glucosidase inhibitor OR Acarbose OR Miglitol OR Voglibose OR Glitazone OR Thiazolidinedione OR Pioglitazone OR Rivoglitazone OR Rosiglitazone) AND (RCT* OR random*)
