# Supplementary material for: Comparison of antidiabetic drugs added to sulfonylurea monotherapy in patients with type 2 diabetes mellitus: A network meta-analysis
Source: PLoS One. 2018 Aug 27;13(8):e0202563. doi: 10.1371/journal.pone.0202563 (PMC6110472; doi:10.1371/journal.pone.0202563)
Supplement: S3 Table — (PDF) [file pone.0202563.s003.pdf]

S3 Table. Number of total participants and participants with events according to safety outcome and study

Author, year	Hypoglycemia		Serious adverse event	
	**Participants**	**Participants with event**	**Participants**	**Participants with event**
Ba 2017[1]	-	-	-	-
Gantz 2017[2]	189	10	189	2
Yang 2015[3]	278	0	278	2
Hermansen 2007[4]	212	11	212	11
Barnett 2013[5]	138	36	138	12
Garber 2008[6]	515	9	515	18
Pratley 2009[7]	500	62	500	24
Chacra 2009[8]	768	97	768	16
Yale 2017[9]	215	29	215	25
Dungan 2016[10]	299	52	299	9
Forst 2015[11]	161	35	161	17
Strojek 2014[12]	596	57	596	58
Hsieh 2011[13]	98	37	-	-
Scheen 2009[14]	1001	173	1001	447
Marre 2009[15]	1041	65	-	-
Seufert 2008[16]	639	86	-	-
Davidson 2007[17]	245	9	-	-
Buse 2004[18]	377	68	377	19
Araki 2015[19]	336	20	336	20
Kobayashi 2014[20]	114	5	114	2
Wolffenbuttel 2000[21]	574	21	-	-
Kaku 2010[22]	264	5	264	8
Zhu 2003[23]	554	42	-	-
Bachmann 2003[24]	372	10	372	40
**Total RCTs**	**23**		**17**	
**Total**	**9486**	**939**	**6335**	**730**
