# Supplementary material for: Comparison of antidiabetic drugs added to sulfonylurea monotherapy in patients with type 2 diabetes mellitus: A network meta-analysis
Source: PLoS One. 2018 Aug 27;13(8):e0202563. doi: 10.1371/journal.pone.0202563 (PMC6110472; doi:10.1371/journal.pone.0202563)
Supplement: S4 Table — (PDF) [file pone.0202563.s004.pdf]

S4 Table. Number of participants by efficacy outcome and study

Author, year	HbA1c	FPG	Body Weight
Ba 2017[1]	256	256	-
Gantz 2017[2]	189	189	189
Yang 2015[3]	279	279	-
Hermansen 2007[4]	206	206	-
Barnett 2013[5]	123	-	-
Garber 2008[6]	408	408	408
Pratley 2009[7]	495	498	488
Chacra 2009[8]	760	764	
Yale 2017[9]	215	215	-
Dungan 2016[10]	299	299	299
Forst 2015[11]	161	-	-
Strojek 2014[12]	592	592	592
Hsieh 2011[13]	100	100	-
Scheen 2009[14]	1001	-	1001
Marre 2009[15]	1041	-	466
Seufert 2008[16]	639	639	-
Davidson 2007[17]	233	233	-
Buse 2004[18]	377	377	377
Araki 2015[19]	312	312	312
Kobayashi 2014[20]	120	120	120
Wolffenbuttel 2000[21]	-	-	-
Kaku 2010[22]	264	264	264
Zhu 2003[23]	530	530	-
Bachmann 2003[24]	330	330	-
**Total RCTs**	**23**	**19**	**11**
**Total participants**	**8930**	**6611**	**4516**

Note: HbA1c, glycated hemoglobin; FPG, fasting plasma glucose.
