# Supplementary material for: Comparison of antidiabetic drugs added to sulfonylurea monotherapy in patients with type 2 diabetes mellitus: A network meta-analysis
Source: PLoS One. 2018 Aug 27;13(8):e0202563. doi: 10.1371/journal.pone.0202563 (PMC6110472; doi:10.1371/journal.pone.0202563)
Supplement: S5 Table — (PDF) [file pone.0202563.s005.pdf]

S5 Table. Numbers of arms, participants, and participants with events by safety outcome and drug

Drug: Sulphonylurea +	Number of	Hypoglycemia	Serious adverse event
SGLT-2i	Arms	3	3
	Participants with event	81	76
	Participants	869	869
DPP-4i	Arms	9	9
	Participants with event	187	70
	Participants	1852	1852
GLP-1	Arms	4	3
	Participants with event	170	23
	Participants	1364	669
TZD	Arms	6	1
	Participants with event	221	217
	Participants	2004	508
Met	Arms	2	1
	Participants with event	55	3
	Participants	383	63
AGI	Arms	3	2
	Participants with event	31	21
	Participants	297	238
Basal	Arms	1	1
	Participants with event	23	6
	Participants	79	79
PLA	Arms	19	14
	Participants with event	171	314
	Participants	2638	2057
**Total number of**			
**Arms**		47	34
**Participants with event**		939	730
**Participants**		9486	6335

Note: SGLT-2i, sodium-glucose co-transporter-2 inhibitor; DPP-4i, dipeptidyl peptidase-4 inhibitor; GLP-1, glucagon-like peptide-1 receptor agonist; AGI, α-glucosidase inhibitor; TZD, thiazolidinedione; Met, metformin; Basal, basal (long acting) insulin, PLA, placebo.
