# Supplementary material for: Comparison of antidiabetic drugs added to sulfonylurea monotherapy in patients with type 2 diabetes mellitus: A network meta-analysis
Source: PLoS One. 2018 Aug 27;13(8):e0202563. doi: 10.1371/journal.pone.0202563 (PMC6110472; doi:10.1371/journal.pone.0202563)
Supplement: S6 Table — (PDF) [file pone.0202563.s006.pdf]

S6 Table. Numbers of arms and participants by efficacy outcome and drug

Drug: Sulphonylurea +	Number of	HbA1c	FPG	Body Weight
SGLT-2i	Arms	3	3	2
	Participants	844	844	698
DPP-4i	Arms	10	8	4
	Participants	1885	1726	842
GLP-1	Arms	4	3	4
	Participants	1364	669	903
TZD	Arms	5	3	2
	Participants	1601	861	740
Met	Arms	2	2	1
	Participants	381	381	61
AGI	Arms	3	3	1
	Participants	273	273	60
Basal	Arms	1	-	
	Participants	79	-	
PLA	Arms	19	16	8
	Participants	2503	1857	1212
**Total number of**				
**Arms**		**47**	**38**	**22**
**Participants**		**8930**	**6611**	**4516**

Note: HbA1c, glycated hemoglobin; FPG, fasting plasma glucose; SGLT-2i, sodium-glucose co-transporter-2 inhibitor; DPP-4i, dipeptidyl peptidase-4 inhibitor; GLP-1, glucagon-like peptide-1 receptor agonist; AGI, α-glucosidase inhibitor; TZD, thiazolidinedione; Met, metformin; Basal, basal (long acting) insulin, PLA, placebo.
