# Supplementary material for: Comparison of antidiabetic drugs added to sulfonylurea monotherapy in patients with type 2 diabetes mellitus: A network meta-analysis
Source: PLoS One. 2018 Aug 27;13(8):e0202563. doi: 10.1371/journal.pone.0202563 (PMC6110472; doi:10.1371/journal.pone.0202563)
Supplement: S7 Table — (PDF) [file pone.0202563.s007.pdf]

S7 Table. Assessment of the risk of bias in individual studies

Study	Sequence Generation	Allocation Concealment	Blinding of Participants and Personnel	Blinding of Outcome Assessment	Incomplete Outcome Data	Selective Reporting	Other bias
Ba 2017[1]	Unclear	Low	Unclear	Unclear	Low	Low	Low
Gantz 2017[2]	Unclear	Low	Low	Low	Low	Low	Low
Yang 2015[3]	Low	Unclear	Low	Low	Low	Low	Low
Hermansen 2007[4]	Unclear	Low	Low	High	Low	High	Low
Barnett 2013[5]	Unclear	Low	Low	Low	Unclear	Low	Low
Garber 2008[6]	Low	Low	Unclear	Unclear	Unclear	High	Low
Pratley 2009[7]	Low	Unclear	Unclear	Unclear	High	Unclear	Low
Chacra 2009[8]	Low	Low	Unclear	Unclear	Unclear	Unclear	Low
Yale 2017[9]	Unclear	Unclear	Low	Unclear	Low	Unclear	Low
Dungan 2016[10]	Low	Unclear	Low	Low	Low	Unclear	Low
Forst 2015[11]	Unclear	Low	Low	High	Unclear	Low	Low
Strojek 2014[12]	Low	Unclear	Low	Low	Low	Low	Low
Hsieh 2011[13]	Low	Unclear	Low	Low	Low	Unclear	Low
Scheen 2009[14]	Unclear	Unclear	Unclear	Unclear	Low	Unclear	Low
Marre 2009[15]	Low	Unclear	Unclear	Unclear	Low	Unclear	Low
Davidson 2007[16]	Low	Unclear	Low	Unclear	Low	Low	Low
Buse 2004 [17]	Low	Low	Unclear	Unclear	Low	High	Low
Araki 2015[18]	Low	Low	Unclear	Unclear	Low	Low	Low
Kobayashi 2014[19]	Low	Low	Low	Low	Unclear	Unclear	Low
Wolffenbuttel 2000[20]	Unclear	Unclear	Unclear	Unclear	Low	Low	Low
Kaku 2010[21]	Low	Low	Unclear	Low	Low	Low	Low
Zhu 2003[22]	Unclear	Unclear	Low	Low	Unclear	Low	Low
Seufert 2008[23]	Low	Unclear	Low	Low	Unclear	Unclear	Low
Bachmann 2003[24]	Unclear	Unclear	Unclear	Unclear	Low	Low	Low

Reference

1. Ba J, Han P, Yuan G, Mo Z, Pan C, Wu F, et al. Randomized trial assessing the safety and efficacy of sitagliptin in Chinese patients with type 2 diabetes mellitus inadequately controlled on sulfonylurea alone or combined with metformin. Journal of Diabetes. 2017;9(7):667-76. doi: 10.1111/1753-0407.12456. PMID: 27502307.
2. Gantz I, Okamoto T, Ito Y, Sato A, Okuyama K, O'Neill EA, et al. A Randomized, Placebo-Controlled Trial Evaluating the Safety and Efficacy of Adding Omarigliptin to Antihyperglycemic Therapies in Japanese Patients with Type 2 Diabetes and Inadequate Glycemic Control. Diabetes therapy : research, treatment and education of diabetes and related disorders. 2017;8(4):793-810. Epub 2017/06/08. doi: 10.1007/s13300-017-0270-7. PMID: 28589493.
3. Yang W, Xing X, Lv X, Li Y, Ma J, Yuan G, et al. Vildagliptin added to sulfonylurea improves glycemic control without hypoglycemia and weight gain in Chinese patients with type 2 diabetes mellitus. J Diabetes. 2015;7(2):174-81. Epub 2014/05/16. doi: 10.1111/1753-0407.12169. PMID: 24823599.
4. Hermansen K, Kipnes M, Luo E, Fanurik D, Khatami H, Stein P, et al. Efficacy and safety of the dipeptidyl peptidase-4 inhibitor, sitagliptin, in patients with type 2 diabetes mellitus inadequately controlled on glimepiride alone or on glimepiride and metformin. Diabetes, obesity & metabolism. 2007;9(5):733-45. doi: 10.1111/j.1463-1326.2007.00744.x. PMID: 17593236.
5. Barnett AH, Huisman H, Jones R, von Eynatten M, Patel S, Woerle HJ. Linagliptin for patients aged 70 years or older with type 2 diabetes inadequately controlled with

common antidiabetes treatments: a randomised, double-blind, placebo-controlled trial. Lancet. 2013;382(9902):1413-23. doi: 10.1016/S0140-6736(13)61500-7. PMID: 23948125.

6. Garber AJ, Foley JE, Banerji MA, Ebeling P, Gudbjornsdottir S, Camisasca RP, et al. Effects of vildagliptin on glucose control in patients with type 2 diabetes inadequately controlled with a sulphonylurea. Diabetes, obesity & metabolism. 2008;10(11):1047-56. doi: 10.1111/j.1463-1326.2008.00859.x. PMID: 18284434.

7. Pratley RE, Kipnes MS, Fleck PR, Wilson C, Mekki Q, Alogliptin Study G. Efficacy and safety of the dipeptidyl peptidase-4 inhibitor alogliptin in patients with type 2 diabetes inadequately controlled by glyburide monotherapy. Diabetes, obesity & metabolism. 2009;11(2):167-76. doi: 10.1111/j.1463-1326.2008.01016.x. PMID: 19125778.

8. Chacra AR, Tan GH, Apanovitch A, Ravichandran S, List J, Chen R. Saxagliptin added to a submaximal dose of sulphonylurea improves glycaemic control compared with uptitration of sulphonylurea in patients with type 2 diabetes: a randomised controlled trial. International journal of clinical practice. Int J Clin Pract. 2009; 63(9):1395-406. doi:10.1111/j.1742-1241.2009.02143.x. PMID: 19614786

9. Yale JF, Xie J, Sherman SE, Garceau C. Canagliflozin in Conjunction With Sulfonylurea Maintains Glycemic Control and Weight Loss Over 52 Weeks: A Randomized, Controlled Trial in Patients With Type 2 Diabetes Mellitus. Clinical therapeutics. 2017;39(11):2230-42.e2. doi: 10.1016/j.clinthera.2017.10.003. PMID: 29103664.

10. Dungan KM, Weitgasser R, Perez Manghi F, Pintilei E, Fahrbach JL, Jiang HH, et al. A 24-week study to evaluate the efficacy and safety of once-weekly dulaglutide added on to glimepiride in type 2 diabetes (AWARD-8). Diabetes, obesity & metabolism. 2016;18(5):475-82. doi: 10.1111/dom.12634. PMID: 26799540.

11. Forst T, Koch C, Dworak M. Vildagliptin versus insulin in patients with type 2 diabetes mellitus inadequately controlled with sulfonylurea: results from a randomized, 24 week study. Current medical research and opinion. 2015; 31(6): 1079-84. doi: 10.1185/03007995.2015.1039936. PMID: 25867771.

12. Strojek K, Yoon KH, Hruba V, Sugg J, Langkilde AM, Parikh S. Dapagliflozin added to glimepiride in patients with type 2 diabetes mellitus sustains glycemic control and weight loss over 48 weeks: a randomized, double-blind, parallel-group, placebo-controlled trial. Diabetes therapy : research, treatment and education of diabetes and related disorders. 2014;5(1):267-83. doi: 10.1007/s13300-014-0072-0. PMID: 24920277.

13. Hsieh SH, Shih KC, Chou CW, Chu CH. Evaluation of the efficacy and tolerability of miglitol in Chinese patients with type 2 diabetes mellitus inadequately controlled by diet and sulfonylureas. Acta diabetologica. 2011;48(1):71-7. doi: 10.1007/s00592-010-0220-6. PMID: 20963449.

14. Scheen AJ, Tan MH, Betteridge DJ, Birkeland K, Schmitz O, Charbonnel B. Long-term glycaemic effects of pioglitazone compared with placebo as add-on treatment to metformin or sulphonylurea monotherapy in PROactive (PROactive 18). Diabet Med. 2009;26(12):1242-9. doi: 10.1111/j.1464-5491.2009.02857.x. PMID: 20002476.

15. Marre M, Shaw J, Brandle M, Bebakar WM, Kamaruddin NA, Strand J, et al. Liraglutide, a once-daily human GLP-1 analogue, added to a sulphonylurea over 26 weeks produces greater improvements in glycaemic and weight control compared with adding rosiglitazone or placebo in subjects with Type 2 diabetes (LEAD-1 SU). Diabet Med. 2009;26(3):268-78. doi: 10.1111/j.1464-5491.2009.02666.x. PMID: 19317822.

16. Davidson JA, McMorn SO, Waterhouse BR, Cobitz AR. A 24-week, multicenter, randomized, double-blind, placebo-controlled, parallel-group study of the efficacy and tolerability of combination therapy with rosiglitazone and sulfonylurea in African American and Hispanic American patients with type 2 diabetes inadequately controlled with sulfonylurea monotherapy. Clinical therapeutics. 2007;29(9):1900-14. doi: 10.1016/j.clinthera.2007.09.011. PMID: 18035190.

17. Buse JB, Henry RR, Han J, Kim DD, Fineman MS, Baron AD, et al. Effects of exenatide (exendin-4) on glycemic control over 30 weeks in sulfonylurea-treated patients with type 2 diabetes. Diabetes Care. 2004;27(11):2628-35. PMID: 15504997.

18. Araki E, Tanizawa Y, Tanaka Y, Taniguchi A, Koiwai K, Kim G, et al. Long-term treatment with empagliflozin as add-on to oral antidiabetes therapy in Japanese patients with type 2 diabetes mellitus. Diabetes, obesity & metabolism. 2015;17(7):665-74. doi: 10.1111/dom.12464. PMID: 25772548.

19. Kobayashi K, Yokoh H, Sato Y, Takemoto M, Uchida D, Kanatsuka A, et al. Efficacy and safety of the dipeptidyl peptidase-4 inhibitor sitagliptin compared with alpha-glucosidase inhibitor in Japanese patients with type 2 diabetes inadequately controlled on sulfonylurea alone (SUCCESS-2): a multicenter, randomized, open-label,

non-inferiority trial. Diabetes, obesity & metabolism [Internet]. 2014; 16(8): 761-5. doi: 10.1111/dom.12264. PMID: 24447683.

20. Wolffenbuttel BH, Gomis R, Squatrito S, Jones NP, Patwardhan RN. Addition of low-dose rosiglitazone to sulphonylurea therapy improves glycaemic control in Type 2 diabetic patients. Diabet Med. 2000;17(1):40-7. PMID: 10691158.

21. Kaku K, Rasmussen MF, Clauson P, Seino Y. Improved glycaemic control with minimal hypoglycaemia and no weight change with the once-daily human glucagon-like peptide-1 analogue liraglutide as add-on to sulphonylurea in Japanese patients with type 2 diabetes. Diabetes, obesity & metabolism. 2010;12(4):341-7. doi: 10.1111/j.1463-1326.2009.01194.x. PMID: 20380655.

22. Zhu XX, Pan CY, Li GW, Shi HL, Tian H, Yang WY, et al. Addition of rosiglitazone to existing sulfonylurea treatment in chinese patients with type 2 diabetes and exposure to hepatitis B or C. Diabetes technology & therapeutics. 2003; 5(1):33-42. doi: 10.1089/152091503763816445. PMID: 12725705.

23. Seufert J, Urquhart R. 2-year effects of pioglitazone add-on to sulfonylurea or metformin on oral glucose tolerance in patients with type 2 diabetes. Diabetes research and clinical practice. 2008;79(3):453-60. doi: 10.1016/j.diabres.2007.11.014. PMID: 18160120.

24. Bachmann W, Petzinna D, Raptis SA, Wascher T, Westermeier T. Long-term improvement of metabolic control by acarbose in type 2 diabetes patients poorly controlled with maximum sulfonylurea therapy. Clin Drug Investig. 2003;23(10):679-86. PMID: 17535083.
