# Supplementary material for: Comparison of antidiabetic drugs added to sulfonylurea monotherapy in patients with type 2 diabetes mellitus: A network meta-analysis
Source: PLoS One. 2018 Aug 27;13(8):e0202563. doi: 10.1371/journal.pone.0202563 (PMC6110472; doi:10.1371/journal.pone.0202563)
Supplement: S8 Table — (PDF) [file pone.0202563.s008.pdf]

S8 Table. Assessment of the loop inconsistency within networks

Loop	Inconsistency factor	95% confidence interval	P-value	Loop heterogeneity tau^2
**HbA1c**				
PLA-SGLT2-TZD-Basal	0.475	0.00-1.76	0.468	0.145
PLA-GLP1-TZD	0.447	0.00-1.40	0.357	0.139
PLA-DPP4-Met	0.017	0.00-0.44	0.939	0.005
**FPG**				
PLA-SGLT2-TZD-AGI	1.493	0.00-3.99	0.241	0.439
PLA-DPP4-Met	0.065	0.00-1.00	0.891	0.007
**Body weight**				
PLA-GLP1-TZD	2.221	0.00-5.47	0.180	0.000
**Hypoglycemia**				
PLA-DPP4-Met	0.915	0.00-2.96	0.380	0.018
PLA-SGLT2-TZD-AGI	0.561	0.00-2.27	0.521	0.083
PLA-GLP1-TZD	0.183	0.00-1.75	0.819	0.184
**Serious adverse event**				
PLA-DPP4-Basal	0.029	0.00-2.94	0.985	0.000

Note: HbA1c, glycated hemoglobin; FPG, fasting plasma glucose; SGLT2, sodium-glucose co-transporter-2 inhibitor; DPP4, dipeptidyl peptidase-4 inhibitor; GLP1, glucagon-like peptide-1 receptor agonist; AGI, α-glucosidase inhibitor; TZD, thiazolidinedione; Met, metformin; Basal, basal (long acting) insulin, PLA, placebo.
