# Supplementary material for: Comparison of antidiabetic drugs added to sulfonylurea monotherapy in patients with type 2 diabetes mellitus: A network meta-analysis
Source: PLoS One. 2018 Aug 27;13(8):e0202563. doi: 10.1371/journal.pone.0202563 (PMC6110472; doi:10.1371/journal.pone.0202563)
Supplement: S9 Table — (PDF) [file pone.0202563.s009.pdf]

S9 Table. Assessment of global inconsistency within networks using the 'design-by-treatment' model

Network outcome	Chi square	P value for test of global inconsistency
**HbA1c**	3.52	0.4745
**FPG**	5.43	0.0663
**Body weight**	1.80	0.1800
**Hypoglycemia**	3.81	0.4329
**Serious adverse event**	0.00	0.9966

Note: HbA1c, glycated hemoglobin; FPG, fasting plasma glucose.
