# Supplementary material for: Comparison of antidiabetic drugs added to sulfonylurea monotherapy in patients with type 2 diabetes mellitus: A network meta-analysis
Source: PLoS One. 2018 Aug 27;13(8):e0202563. doi: 10.1371/journal.pone.0202563 (PMC6110472; doi:10.1371/journal.pone.0202563)
Supplement: S10 Table — (PDF) [file pone.0202563.s010.pdf]

S10 Table. Contributions of direct evidence in the entire network

Outcomes	Drug A	Drug B	No. of trials	Contribution to the network, %
**HbA1c**	SGLT-2i	PLA	2	14.3
	DPP-4i	PLA	8	17.8
	GLP-1	PLA	4	11.2
	TZD	PLA	4	2.8
	AGI	PLA	2	6.3
	SGLT-2i	Met	1	11.4
	DPP-4i	AGI	1	5.3
	DPP-4i	Basal	1	9.9
	GLP-1	TZD	1	10.8
	TZD	Met	1	10.3
**FPG**	SGLT-2i	PLA	2	20.9
	DPP-4i	PLA	7	15.1
	GLP-1	PLA	3	12.9
	TZD	PLA	2	5.8
	AGI	PLA	2	9.2
	SGLT-2i	Met	1	18.7
	DPP-4i	AGI	1	4.2
	TZD	Met	1	13.2
**Body weight**	SGLT-2i	PLA	1	21.0
	DPP-4i	PLA	3	21.0
	GLP-1	PLA	3	19.7
	TZD	PLA	1	1.6
	SGLT-2i	Met	1	12.6
	DPP-4i	AGI	1	12.6
	GLP-1	TZD	1	11.6
**Hypoglycemia**	SGLT-2i	PLA	2	11.3
	DPP-4i	PLA	7	20.1
	GLP-1	PLA	4	9.0
	TZD	PLA	5	13.0
	AGI	PLA	2	5.6
	SGLT-2i	Met	1	5.6
	DPP-4i	AGI	1	5.5
	DPP-4i	Basal	1	10.6
	GLP-1	TZD	1	8.1
	TZD	Met	1	11.2
**Serious adverse event**	SGLT-2i	PLA	2	20.2
	DPP-4i	PLA	7	19.9
	GLP-1	PLA	3	11.8
	TZD	PLA	1	11.8
	AGI	PLA	1	11.6

	SGLT-2i	Met	1	11.8
	DPP-4i	AGI	1	1.2
	DPP-4i	Basal	1	11.8

Note: HbA1c, glycated hemoglobin; FPG, fasting plasma glucose; SGLT-2i, sodium-glucose co-transporter-2 inhibitor; DPP-4i, dipeptidyl peptidase-4 inhibitor; GLP-1, glucagon-like peptide-1 receptor agonist; AGI, α-glucosidase inhibitor; TZD, thiazolidinedione; Met, metformin; Basal, basal (long acting) insulin, PLA, placebo.
