# Supplementary material for: Comparison of antidiabetic drugs added to sulfonylurea monotherapy in patients with type 2 diabetes mellitus: A network meta-analysis
Source: PLoS One. 2018 Aug 27;13(8):e0202563. doi: 10.1371/journal.pone.0202563 (PMC6110472; doi:10.1371/journal.pone.0202563)
Supplement: S11 Table — (PDF) [file pone.0202563.s011.pdf]

S11 Table. Pairwise random-effects meta-analyses of hypoglycemia and serious adverse events

Outcomes	Drug A	Drug B	No. of trials	No. of participants	No. of participants with event	Odds Ratio (95% CI)	I^2 (%)	Tau^2
Hypoglycemia	SGLT-2i	PLA	2	811	86	1.23 [0.70, 2.18]	10	0.02
	SGLT-2i	Met	1	366	20	0.67 [0.24, 1.93]	/	/
	DPP-4i	PLA	7	2600	225	1.54 [1.10, 2.15]	0	0
	DPP-4i	Basal	1	161	35	0.42 [0.19, 0.91]	/	/
	DPP-4i	AGI	1	114	5	0.61 [0.10, 3.78]	/	/
	GLP-1	PLA	3	940	125	7.56 [3.44, 16.59]	0	0
	GLP-1	TZD	1	927	62	1.80 [0.90, 3.59]	/	/
	TZD	PLA	4	2374	245	2.87 [1.21, 6.81]	50	0.36
	TZD	Met	1	639	86	0.69 [0.43, 1.09]	/	
	AGI	PLA	2	470	47	1.49 [0.24, 9.26]	77	1.36
Serious adverse event	SGLT-2i	PLA	2	811	83	0.84 [0.42, 1.67]	41	0.1
	SGLT-2i	Met	1	336	20	1.33 [0.38, 4.68]	/	/
	DPP-4i	PLA	7	2600	85	0.94 [0.57, 1.55]	0	0
	DPP-4i	Basal	1	161	17	1.88 [0.66, 5.37]	/	/
	DPP-4i	AGI	1	114	2	0.93 [0.06, 15.26]	/	/
	GLP-1	PLA	3	940	36	0.71 [0.24, 2.08]	35	0.34
	TZD	PLA	1	1001	447	0.85 [0.66, 1.09]	/	/
	AGI	PLA	1	372	40	1.04 [0.54, 2.00]	/	/

Note: SGLT-2i, sodium-glucose co-transporter-2 inhibitor; DPP-4i, dipeptidyl peptidase-4 inhibitor; GLP-1, glucagon-like peptide-1 receptor agonist; AGI, α-glucosidase inhibitor; TZD, thiazolidinedione; Met, metformin; Basal, basal (long acting) insulin, PLA, placebo.
