# Supplementary material for: Comparison of antidiabetic drugs added to sulfonylurea monotherapy in patients with type 2 diabetes mellitus: A network meta-analysis
Source: PLoS One. 2018 Aug 27;13(8):e0202563. doi: 10.1371/journal.pone.0202563 (PMC6110472; doi:10.1371/journal.pone.0202563)
Supplement: S12 Table — (PDF) [file pone.0202563.s012.pdf]

S12 Table. Pairwise random-effects meta-analyses of HbA1c, fasting plasma glucose, and body weight

Outcomes	Drug A	Drug B	No. of trials	No. of participants	Mean Difference (95% CI)	I^2 (%)	Tau^2
**HbA1c (%)**	SGLT-2i	PLA	2	807	-0.55 (-0.71, -0.39)	0	0
	SGLT-2i	Met	1	312	0.02 (-0.15, 0.19)	/	/
	DPP-4i	PLA	8	2716	-0.68 (-0.77, -0.60)	49	0.01
	DPP-4i	Basal	1	161	0.30 (0.05, 0.55)	/	/
	DPP-4i	AGI	1	120	-0.10 (-0.34, 0.14)	/	/
	GLP-1	PLA	3	940	-1.05 (-1.35, -0.76)	74	0.05
	GLP-1	TZD	1	927	-0.49 (-0.64, -0.34)	/	/
	TZD	PLA	3	1877	-1.04 (-1.64, -0.44)	95	0.27
	TZD	Met	1	639	0.13 (-0.06, 0.32)	/	/
	AGI	PLA	2	430	-0.60 (-0.83, -0.37)	0	0
**FPG (mmol/l)**	SGLT-2i	PLA	2	807	-1.56 (-2.20, -0.93)	61	0.13
	SGLT-2i	Met	1	312	-0.44 (-0.73, -0.15)	/	/
	DPP-4i	PLA	7	2600	-0.62 (-0.78, -0.47)	29	0.01
	DPP-4i	AGI	1	120	0.11 (-0.64, 0.86)	/	/
	GLP-1	PLA	3	940	-1.46 (-2.04, -0.88)	57	0.15
	TZD	PLA	2	763	-2.77 (-4.14, -1.40)	88	0.86
	TZD	Met	1	639	-0.10 (-0.55, 0.35)	/	/
	AGI	PLA	2	430	-0.78 (-1.29, -0.28)	0	0
**Body weight (kg)**	SGLT-2i	PLA	1	592	-1.00 (-1.73, -0.27)	/	/
	SGLT-2i	Met	1	312	-2.45 (-3.10, -1.80)	/	/
	DPP-4i	PLA	3	1085	1.00 (0.90, 1.11)	0	0
	DPP-4i	AGI	1	120	1.30 (0.62, 1.98)	/	/
	GLP-1	PLA	3	940	-0.62 (-1.17, -0.08)	0	0
	TZD	Met	1	466	-2.30 (-3.20, -1.40)	/	/

Note: HbA1c, glycated hemoglobin; FPG, fasting plasma glucose; SGLT-2i, sodium-glucose co-transporter-2 inhibitor; DPP-4i, dipeptidyl peptidase-4 inhibitor; GLP-1, glucagon-like peptide-1 receptor agonist; AGI, α-glucosidase inhibitor; TZD, thiazolidinedione; Met, metformin; Basal, basal (long acting) insulin, PLA, placebo.
