# Supplementary material for: Comparison of antidiabetic drugs added to sulfonylurea monotherapy in patients with type 2 diabetes mellitus: A network meta-analysis
Source: PLoS One. 2018 Aug 27;13(8):e0202563. doi: 10.1371/journal.pone.0202563 (PMC6110472; doi:10.1371/journal.pone.0202563)
Supplement: S1 Fig — (PDF) [file pone.0202563.s013.pdf]

S1 Fig. The distribution of study-level characteristics according to drug class

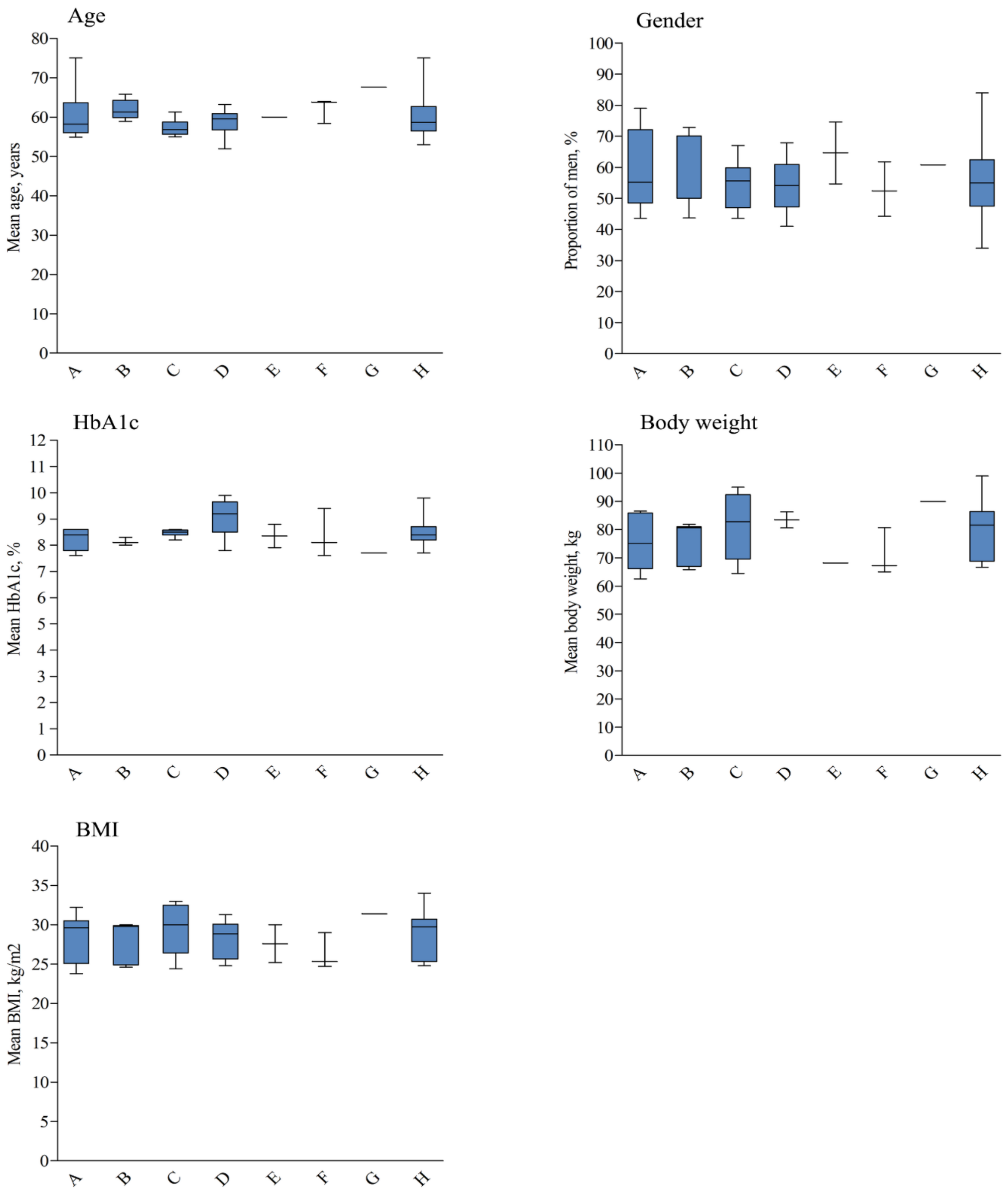

Note: A, dipeptidyl peptidase-4 inhibitor; B, sodium-glucose co-transporter-2 inhibitor; C, glucagon-like peptide-1 receptor agonist; D, thiazolidinedione; E, metformin; F, α-glucosidase inhibitor; G, basal (long acting) insulin; H, placebo. The limits of the boxes represent the 25th and 75th centile, the middle line represents the median, and the error bars show the adjacent values.
