# Supplementary figures and images for: Comparison of antidiabetic drugs added to sulfonylurea monotherapy in patients with type 2 diabetes mellitus: A network meta-analysis
Source: PLoS One. 2018 Aug 27;13(8):e0202563. doi: 10.1371/journal.pone.0202563 (PMC6110472; doi:10.1371/journal.pone.0202563)

**S2 Figure.** Cochrane system bias evaluation chart of eligible studies

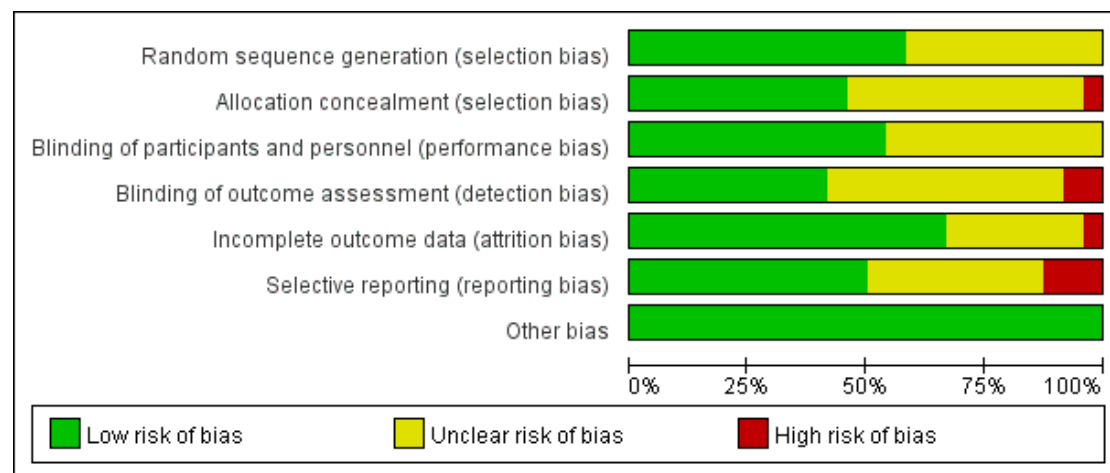

Supplement: S2 Fig — (PDF) [file pone.0202563.s014.pdf]

**S3 Figure.** Comparison-adjusted funnel plot for efficacy and safety outcomes

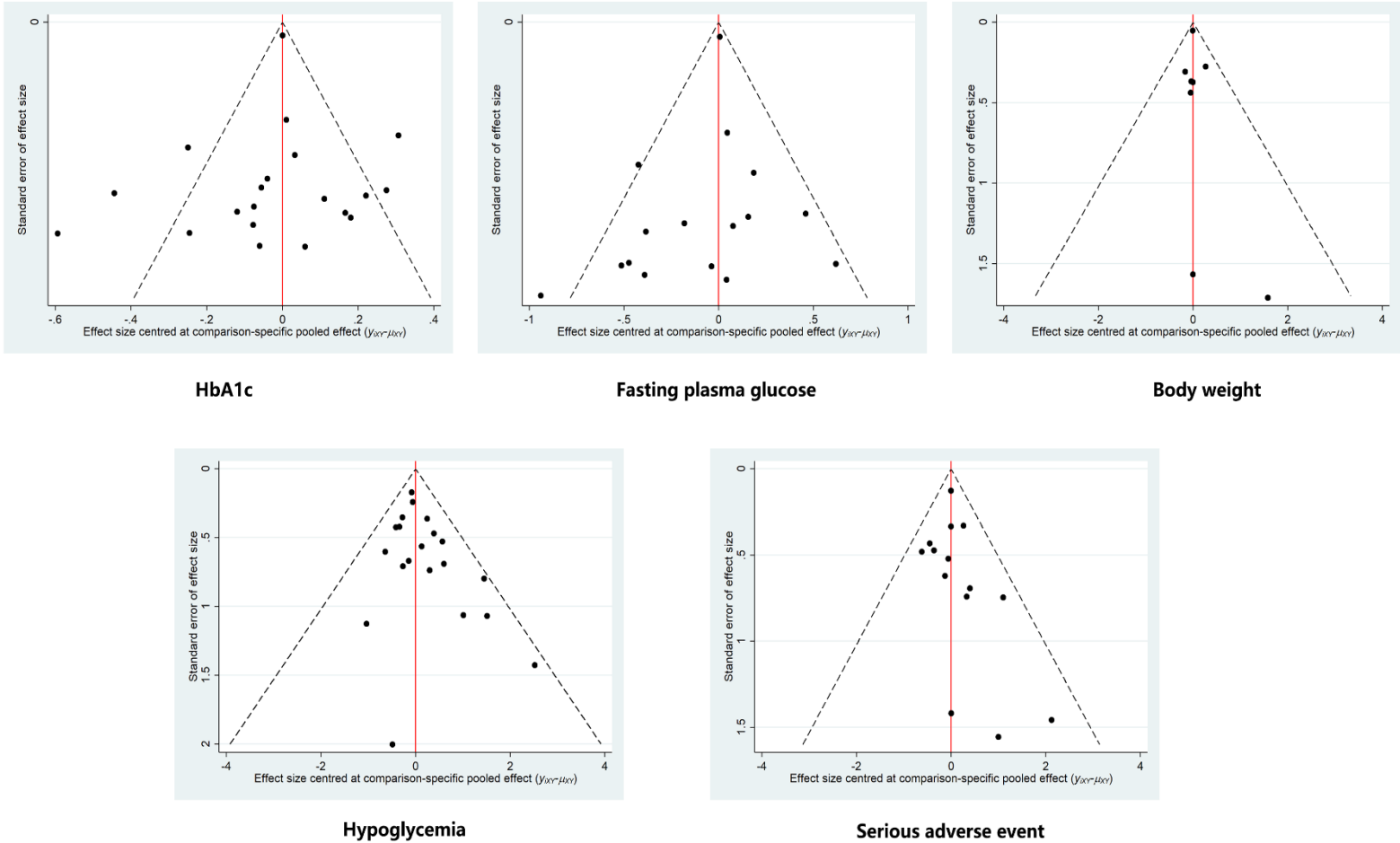

Supplement: S3 Fig — (PDF) [file pone.0202563.s015.pdf]
